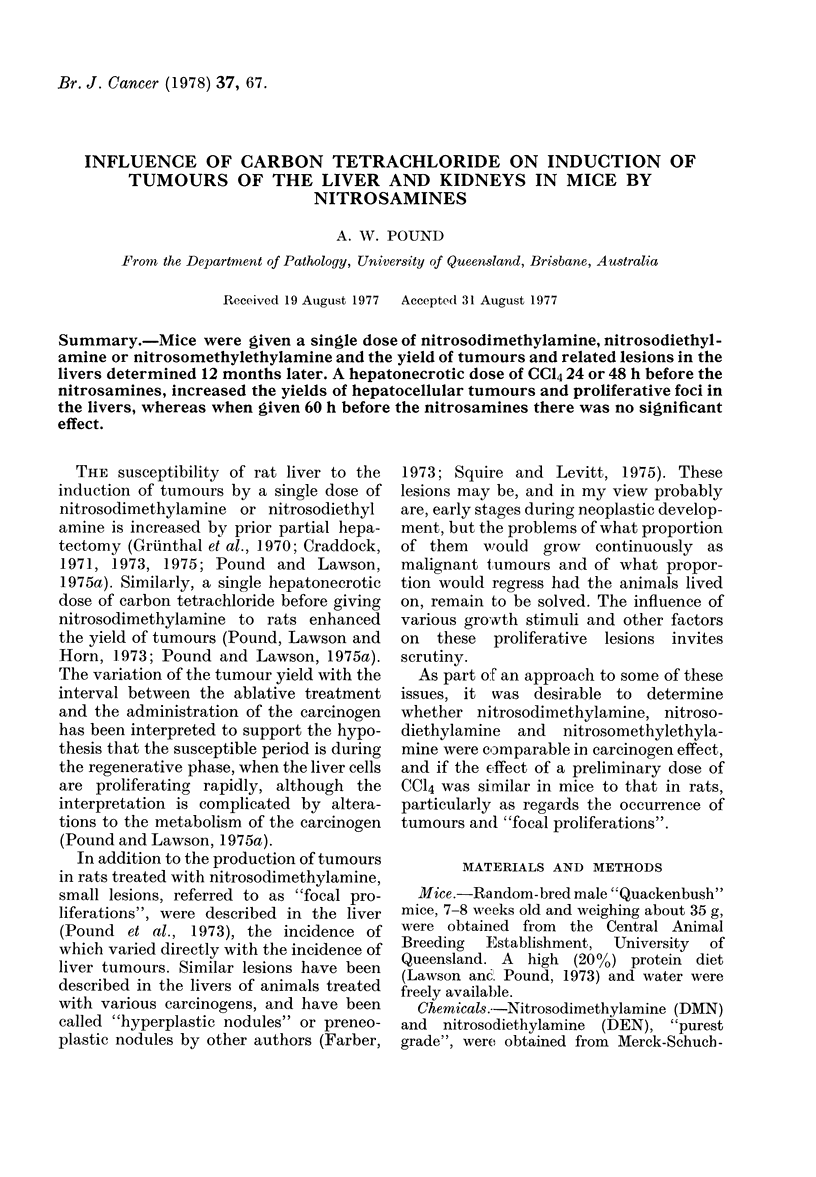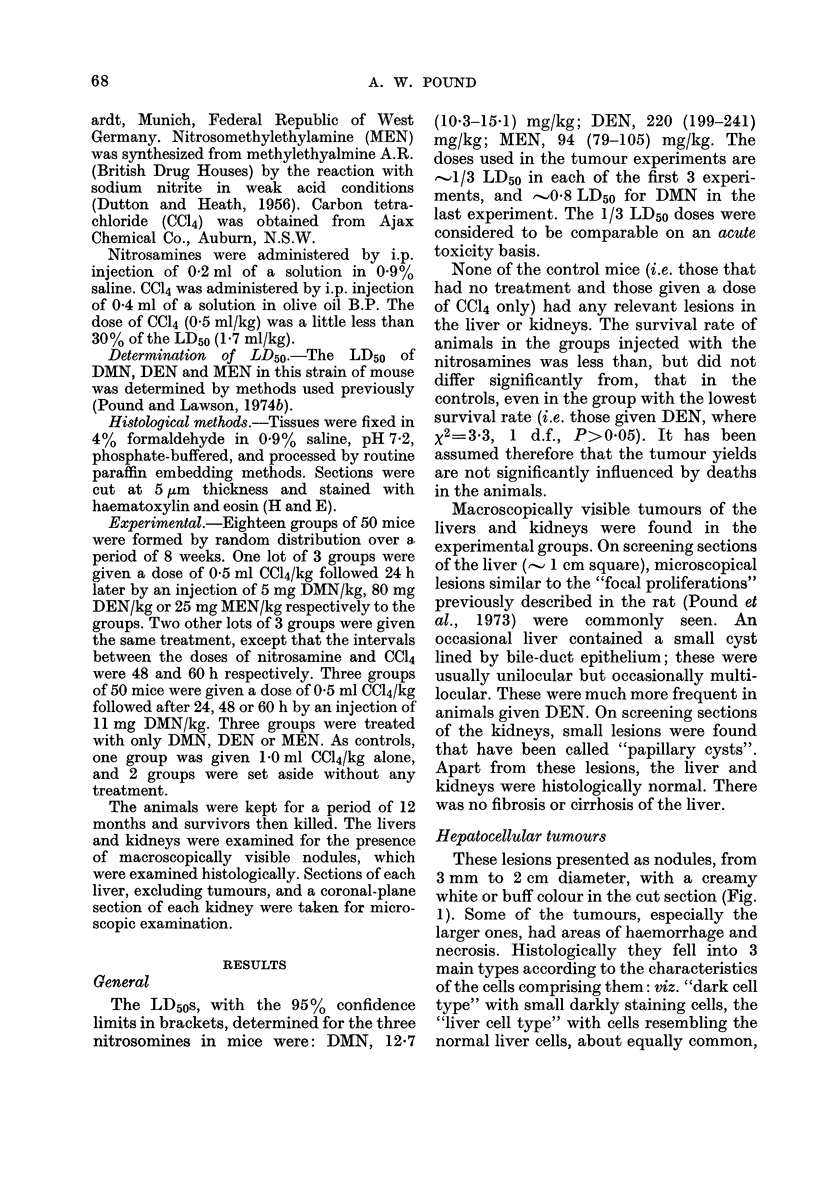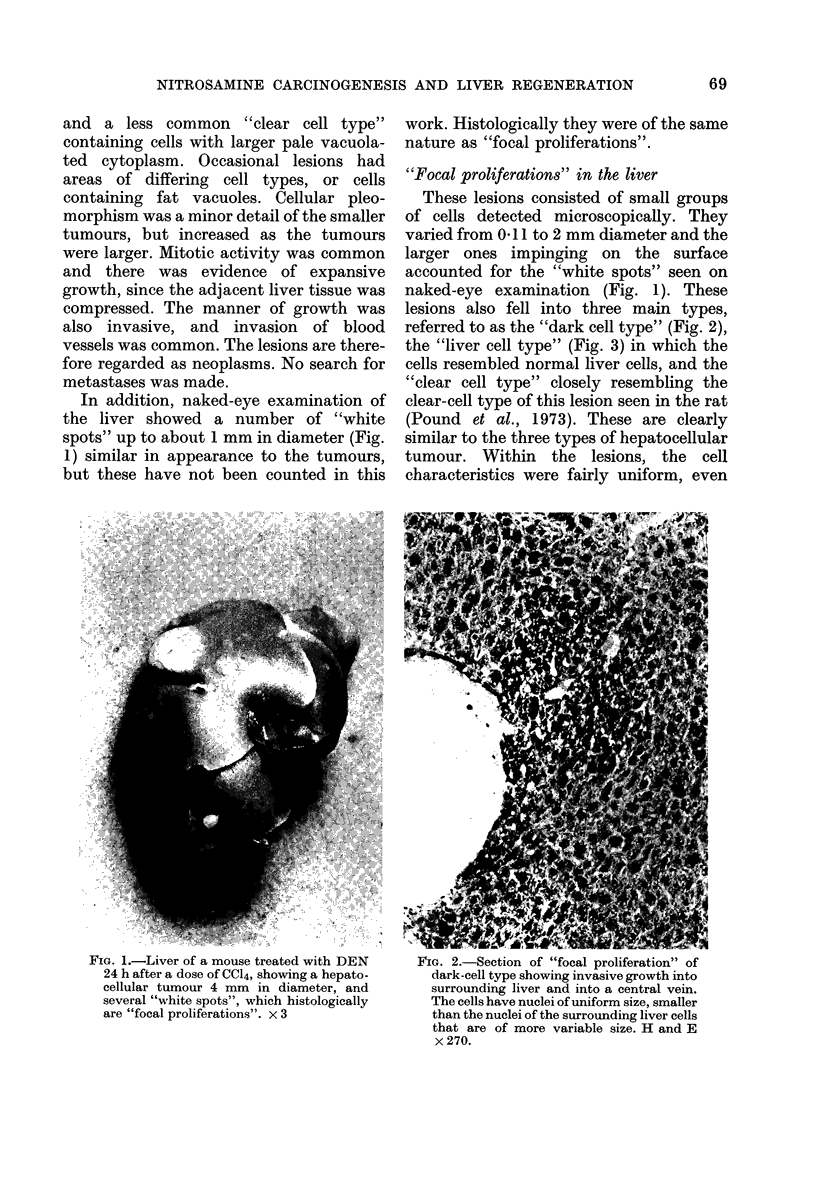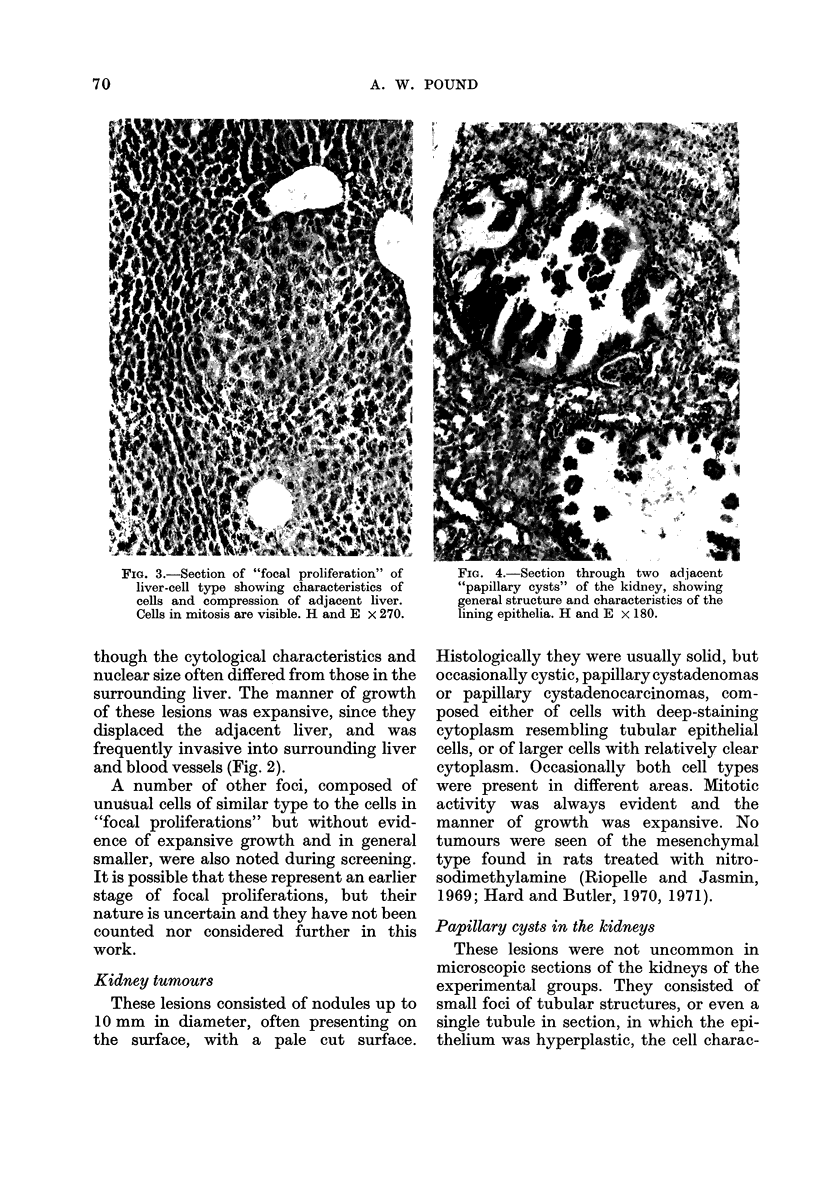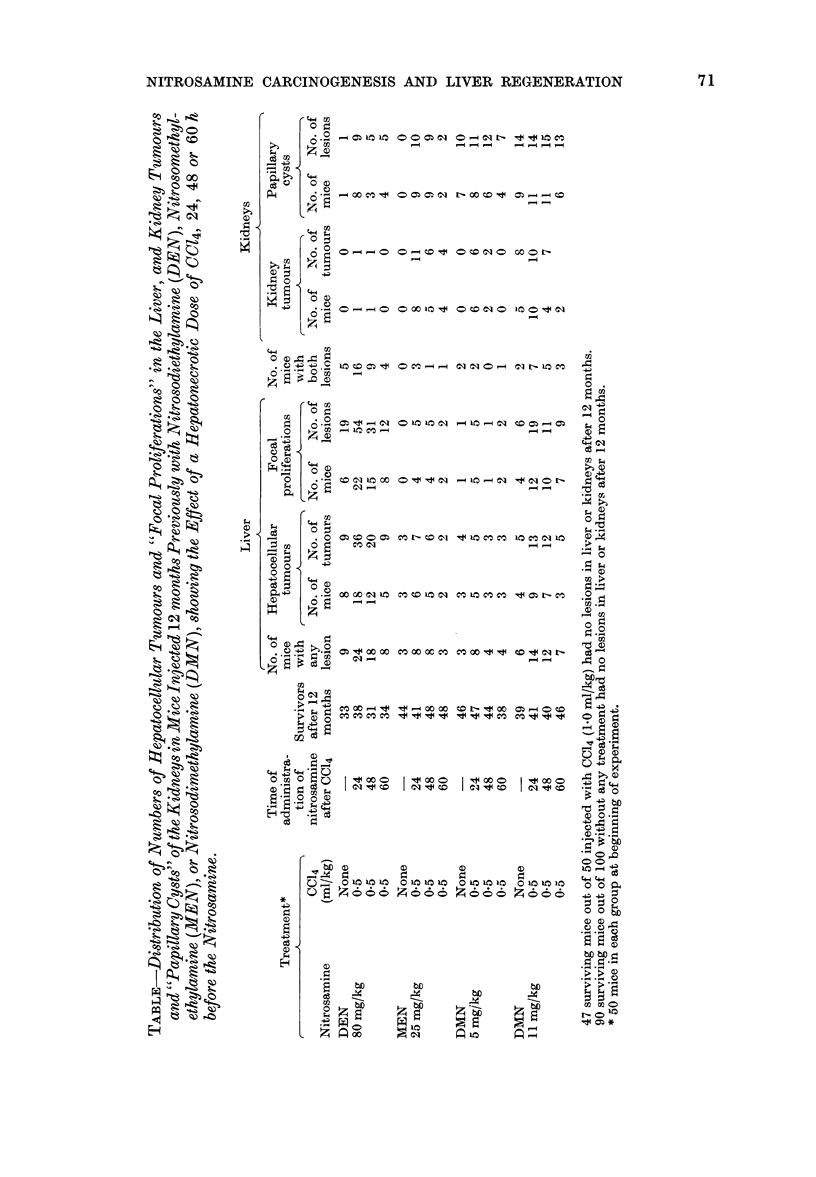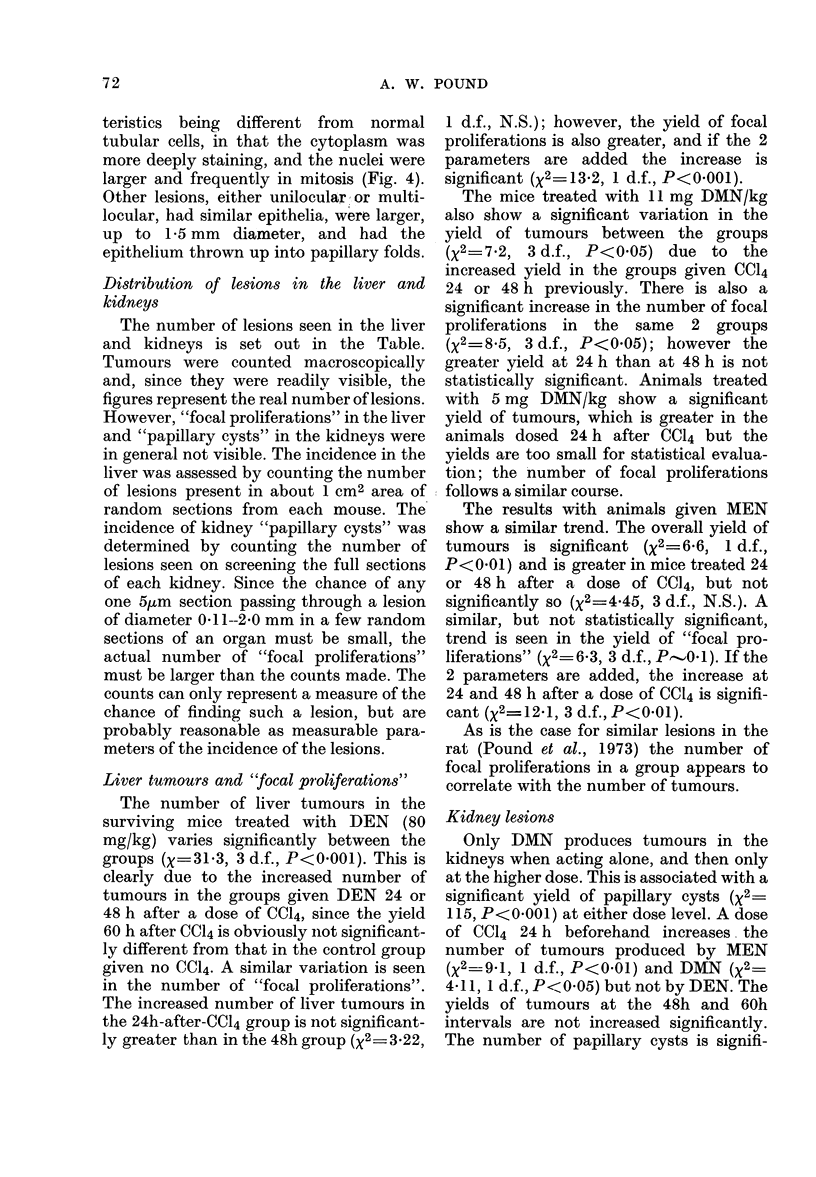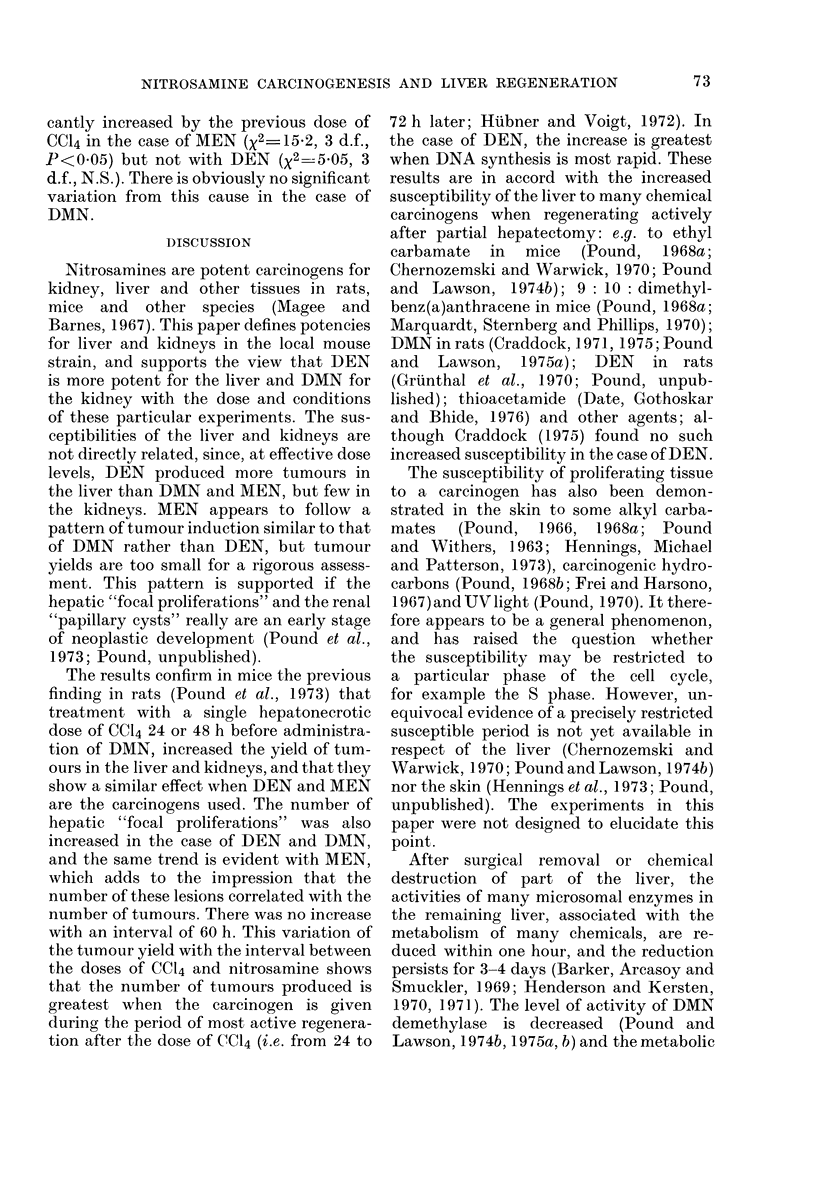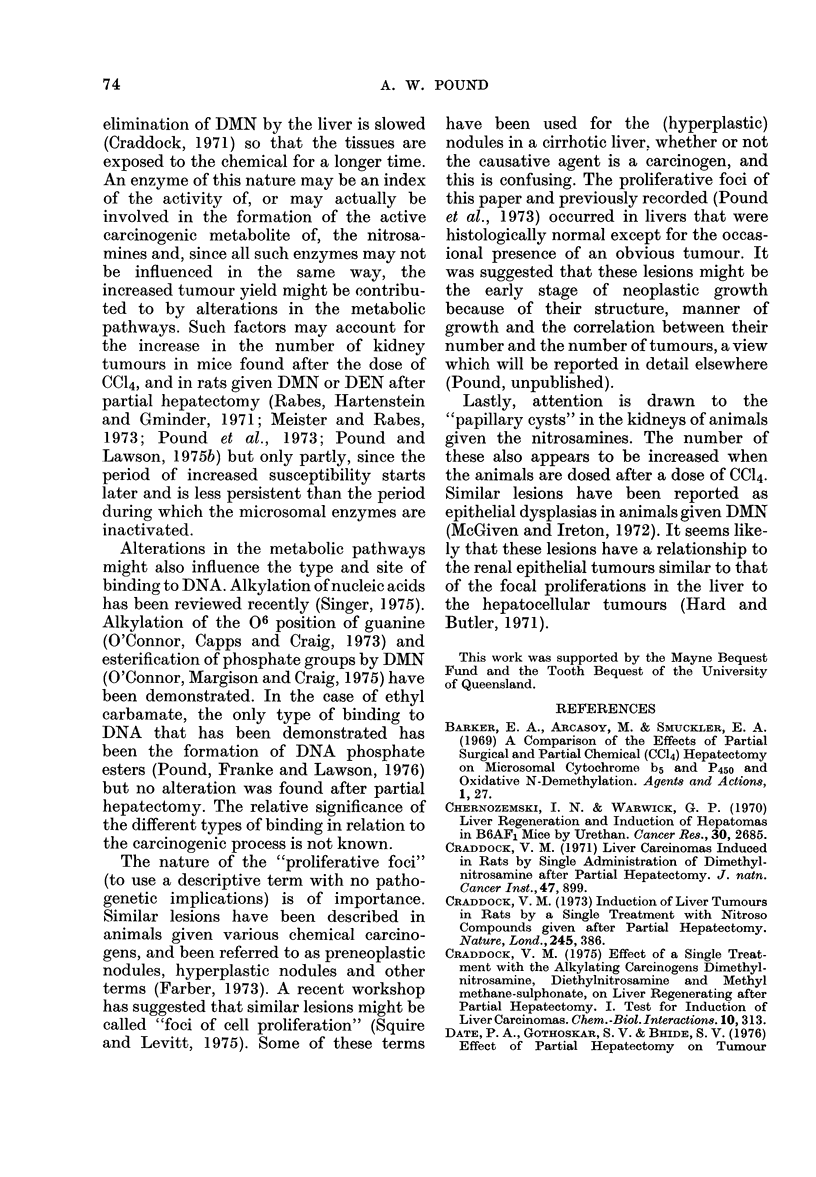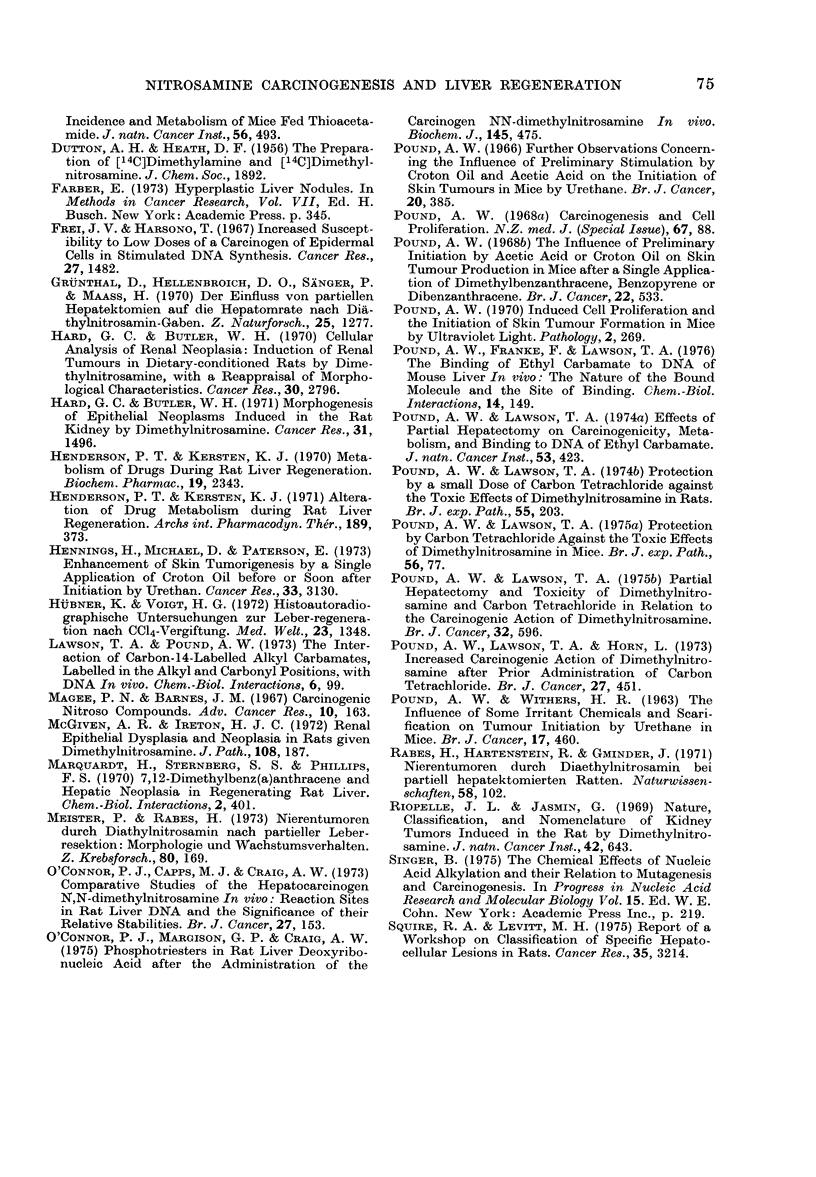# Influence of carbon tetrachloride on induction of tumours of the liver and kidneys in mice by netrosamines.

**DOI:** 10.1038/bjc.1978.10

**Published:** 1978-01

**Authors:** A. W. Pound

## Abstract

**Images:**


*Br. J. Cancer* (1978) **37**, 67.

### INFLUENCE OF CARBON TETRACHLORIDE ON INDUCTION OF TUMOURS OF THE LIVER AND KIDNEYS IN MICE BY NITROSAMINES

A. W. POUND

*From the Department of Pathology, University of Queensland, Brisbane, Australia*

Received 19 August 1977 Accepted 31 August 1977

**Summary.—Mice were given a single dose of nitrosodimethylamine, nitrosodiethylamine or nitrosomethylethylamine and the yield of tumours and related lesions in the livers determined 12 months later. A hepatonecrotic dose of $CCl_4$ 24 or 48 h before the nitrosamines, increased the yields of hepatocellular tumours and proliferative foci in the livers, whereas when given 60 h before the nitrosamines there was no significant effect.**

THE susceptibility of rat liver to the induction of tumours by a single dose of nitrosodimethylamine or nitrosodiethyl amine is increased by prior partial hepatectomy (Grünthal *et al.*, 1970; Craddock, 1971, 1973, 1975; Pound and Lawson, 1975*a*). Similarly, a single hepatonecrotic dose of carbon tetrachloride before giving nitrosodimethylamine to rats enhanced the yield of tumours (Pound, Lawson and Horn, 1973; Pound and Lawson, 1975*a*). The variation of the tumour yield with the interval between the ablative treatment and the administration of the carcinogen has been interpreted to support the hypothesis that the susceptible period is during the regenerative phase, when the liver cells are proliferating rapidly, although the interpretation is complicated by alterations to the metabolism of the carcinogen (Pound and Lawson, 1975*a*).

In addition to the production of tumours in rats treated with nitrosodimethylamine, small lesions, referred to as "focal proliferations", were described in the liver (Pound *et al.*, 1973), the incidence of which varied directly with the incidence of liver tumours. Similar lesions have been described in the livers of animals treated with various carcinogens, and have been called "hyperplastic nodules" or preneoplastic nodules by other authors (Farber, 1973; Squire and Levitt, 1975). These lesions may be, and in my view probably are, early stages during neoplastic development, but the problems of what proportion of them would grow continuously as malignant tumours and of what proportion would regress had the animals lived on, remain to be solved. The influence of various growth stimuli and other factors on these proliferative lesions invites scrutiny.

As part of an approach to some of these issues, it was desirable to determine whether nitrosodimethylamine, nitrosodiethylamine and nitrosomethylethylamine were comparable in carcinogen effect, and if the effect of a preliminary dose of $CCl_4$ was similar in mice to that in rats, particularly as regards the occurrence of tumours and "focal proliferations".

### MATERIALS AND METHODS

*Mice.*—Random-bred male "Quackenbush" mice, 7–8 weeks old and weighing about 35 g, were obtained from the Central Animal Breeding Establishment, University of Queensland. A high (20%) protein diet (Lawson and Pound, 1973) and water were freely available.

*Chemicals.*—Nitrosodimethylamine (DMN) and nitrosodiethylamine (DEN), "purest grade", were obtained from Merck-Schuchardt, Munich, Federal Republic of West Germany. Nitrosomethylethylamine (MEN) was synthesized from methylethyalmine A.R. (British Drug Houses) by the reaction with sodium nitrite in weak acid conditions (Dutton and Heath, 1956). Carbon tetrachloride ($CCl_4$) was obtained from Ajax Chemical Co., Auburn, N.S.W.

Nitrosamines were administered by i.p. injection of 0·2 ml of a solution in 0·9% saline. $CCl_4$ was administered by i.p. injection of 0·4 ml of a solution in olive oil B.P. The dose of $CCl_4$ (0·5 ml/kg) was a little less than 30% of the $LD_{50}$ (1·7 ml/kg).

*Determination of $LD_{50}$.*—The $LD_{50}$ of DMN, DEN and MEN in this strain of mouse was determined by methods used previously (Pound and Lawson, 1974*b*).

*Histological methods.*—Tissues were fixed in 4% formaldehyde in 0·9% saline, pH 7·2, phosphate-buffered, and processed by routine paraffin embedding methods. Sections were cut at 5 $\mu$m thickness and stained with haematoxylin and eosin (H and E).

*Experimental.*—Eighteen groups of 50 mice were formed by random distribution over a period of 8 weeks. One lot of 3 groups were given a dose of 0·5 ml $CCl_4$/kg followed 24 h later by an injection of 5 mg DMN/kg, 80 mg DEN/kg or 25 mg MEN/kg respectively to the groups. Two other lots of 3 groups were given the same treatment, except that the intervals between the doses of nitrosamine and $CCl_4$ were 48 and 60 h respectively. Three groups of 50 mice were given a dose of 0·5 ml $CCl_4$/kg followed after 24, 48 or 60 h by an injection of 11 mg DMN/kg. Three groups were treated with only DMN, DEN or MEN. As controls, one group was given 1·0 ml $CCl_4$/kg alone, and 2 groups were set aside without any treatment.

The animals were kept for a period of 12 months and survivors then killed. The livers and kidneys were examined for the presence of macroscopically visible nodules, which were examined histologically. Sections of each liver, excluding tumours, and a coronal-plane section of each kidney were taken for microscopic examination.

### RESULTS

### General

The $LD_{50}$s, with the 95% confidence limits in brackets, determined for the three nitrosomines in mice were: DMN, 12·7 (10·3–15·1) mg/kg; DEN, 220 (199–241) mg/kg; MEN, 94 (79–105) mg/kg. The doses used in the tumour experiments are $\sim$1/3 $LD_{50}$ in each of the first 3 experiments, and $\sim$0·8 $LD_{50}$ for DMN in the last experiment. The 1/3 $LD_{50}$ doses were considered to be comparable on an *acute* toxicity basis.

None of the control mice (*i.e.* those that had no treatment and those given a dose of $CCl_4$ only) had any relevant lesions in the liver or kidneys. The survival rate of animals in the groups injected with the nitrosamines was less than, but did not differ significantly from, that in the controls, even in the group with the lowest survival rate (*i.e.* those given DEN, where $\chi^2=3·3$, 1 d.f., $P>0·05$). It has been assumed therefore that the tumour yields are not significantly influenced by deaths in the animals.

Macroscopically visible tumours of the livers and kidneys were found in the experimental groups. On screening sections of the liver ($\sim$ 1 cm square), microscopical lesions similar to the "focal proliferations" previously described in the rat (Pound *et al.*, 1973) were commonly seen. An occasional liver contained a small cyst lined by bile-duct epithelium; these were usually unilocular but occasionally multilocular. These were much more frequent in animals given DEN. On screening sections of the kidneys, small lesions were found that have been called "papillary cysts". Apart from these lesions, the liver and kidneys were histologically normal. There was no fibrosis or cirrhosis of the liver.

### *Hepatocellular tumours*

These lesions presented as nodules, from 3 mm to 2 cm diameter, with a creamy white or buff colour in the cut section (Fig. 1). Some of the tumours, especially the larger ones, had areas of haemorrhage and necrosis. Histologically they fell into 3 main types according to the characteristics of the cells comprising them: *viz.* "dark cell type" with small darkly staining cells, the "liver cell type" with cells resembling the normal liver cells, about equally common, and a less common "clear cell type" containing cells with larger pale vacuolated cytoplasm. Occasional lesions had areas of differing cell types, or cells containing fat vacuoles. Cellular pleomorphism was a minor detail of the smaller tumours, but increased as the tumours were larger. Mitotic activity was common and there was evidence of expansive growth, since the adjacent liver tissue was compressed. The manner of growth was also invasive, and invasion of blood vessels was common. The lesions are therefore regarded as neoplasms. No search for metastases was made.

In addition, naked-eye examination of the liver showed a number of "white spots" up to about 1 mm in diameter (Fig. 1) similar in appearance to the tumours, but these have not been counted in this work. Histologically they were of the same nature as "focal proliferations".

*"Focal proliferations" in the liver*

These lesions consisted of small groups of cells detected microscopically. They varied from 0·11 to 2 mm diameter and the larger ones impinging on the surface accounted for the "white spots" seen on naked-eye examination (Fig. 1). These lesions also fell into three main types, referred to as the "dark cell type" (Fig. 2), the "liver cell type" (Fig. 3) in which the cells resembled normal liver cells, and the "clear cell type" closely resembling the clear-cell type of this lesion seen in the rat (Pound *et al.*, 1973). These are clearly similar to the three types of hepatocellular tumour. Within the lesions, the cell characteristics were fairly uniform, even

FIG. 1.—Liver of a mouse treated with DEN 24 h after a dose of $CCl_4$, showing a hepatocellular tumour 4 mm in diameter, and several "white spots", which histologically are "focal proliferations". ×3

FIG. 2.—Section of "focal proliferation" of dark-cell type showing invasive growth into surrounding liver and into a central vein. The cells have nuclei of uniform size, smaller than the nuclei of the surrounding liver cells that are of more variable size. H and E ×270.

FIG. 3.—Section of "focal proliferation" of liver-cell type showing characteristics of cells and compression of adjacent liver. Cells in mitosis are visible. H and E ×270.

FIG. 4.—Section through two adjacent "papillary cysts" of the kidney, showing general structure and characteristics of the lining epithelia. H and E ×180.

though the cytological characteristics and nuclear size often differed from those in the surrounding liver. The manner of growth of these lesions was expansive, since they displaced the adjacent liver, and was frequently invasive into surrounding liver and blood vessels (Fig. 2).

A number of other foci, composed of unusual cells of similar type to the cells in "focal proliferations" but without evidence of expansive growth and in general smaller, were also noted during screening. It is possible that these represent an earlier stage of focal proliferations, but their nature is uncertain and they have not been counted nor considered further in this work.

*Kidney tumours*

These lesions consisted of nodules up to 10 mm in diameter, often presenting on the surface, with a pale cut surface. Histologically they were usually solid, but occasionally cystic, papillary cystadenomas or papillary cystadenocarcinomas, composed either of cells with deep-staining cytoplasm resembling tubular epithelial cells, or of larger cells with relatively clear cytoplasm. Occasionally both cell types were present in different areas. Mitotic activity was always evident and the manner of growth was expansive. No tumours were seen of the mesenchymal type found in rats treated with nitrosodimethylamine (Riopelle and Jasmin, 1969; Hard and Butler, 1970, 1971).

*Papillary cysts in the kidneys*

These lesions were not uncommon in microscopic sections of the kidneys of the experimental groups. They consisted of small foci of tubular structures, or even a single tubule in section, in which the epithelium was hyperplastic, the cell charac-

TABLE—*Distribution of Numbers of Hepatocellular Tumours and "Focal Proliferations" in the Liver, and Kidney Tumours and "Papillary Cysts" of the Kidneys in Mice Injected* 12 *months Previously with Nitrosodiethylamine (DEN), Nitrosomethylethylamine (MEN), or Nitrosodimethylamine (DMN), showing the Effect of a Hepatonecrotic Dose of* $CCl_4$, 24, 48 *or* 60 *h before the Nitrosamine.*

| Treatment* | | | | Liver | | | | | | Kidneys | | | |
|---|---|---|---|---|---|---|---|---|---|---|---|---|---|
| | | Time of administration of nitrosamine after $CCl_4$ | Survivors after 12 months | No. of mice with any lesion | Hepatocellular tumours | | Focal proliferations | | No. of mice with both lesions | Kidney tumours | | Papillary cysts | |
| Nitrosamine | $CCl_4$ (ml/kg) | | | | No. of mice | No. of tumours | No. of mice | No. of lesions | | No. of mice | No. of tumours | No. of mice | No. of lesions |
| DEN | None | — | 33 | 9 | 8 | 9 | 6 | 19 | 5 | 0 | 0 | 1 | 1 |
| 80 mg/kg | 0·5 | 24 | 38 | 24 | 18 | 36 | 22 | 54 | 16 | 1 | 1 | 8 | 9 |
| | 0·5 | 48 | 31 | 18 | 12 | 20 | 15 | 31 | 9 | 1 | 1 | 3 | 5 |
| | 0·5 | 60 | 34 | 8 | 5 | 9 | 8 | 12 | 4 | 0 | 0 | 4 | 5 |
| MEN | None | — | 44 | 3 | 3 | 3 | 0 | 0 | 0 | 0 | 0 | 0 | 0 |
| 25 mg/kg | 0·5 | 24 | 41 | 8 | 6 | 7 | 4 | 5 | 3 | 8 | 11 | 9 | 10 |
| | 0·5 | 48 | 48 | 8 | 5 | 6 | 4 | 5 | 1 | 5 | 6 | 9 | 9 |
| | 0·5 | 60 | 48 | 3 | 2 | 2 | 2 | 2 | 1 | 4 | 4 | 2 | 2 |
| DMN | None | — | 46 | 3 | 3 | 4 | 1 | 1 | 2 | 0 | 0 | 7 | 10 |
| 5 mg/kg | 0·5 | 24 | 47 | 8 | 5 | 5 | 5 | 5 | 2 | 6 | 6 | 8 | 11 |
| | 0·5 | 48 | 44 | 4 | 3 | 3 | 1 | 1 | 0 | 2 | 2 | 6 | 12 |
| | 0·5 | 60 | 38 | 4 | 3 | 3 | 2 | 2 | 1 | 0 | 0 | 4 | 7 |
| DMN | None | — | 39 | 6 | 4 | 5 | 4 | 6 | 2 | 5 | 8 | 9 | 14 |
| 11 mg/kg | 0·5 | 24 | 41 | 14 | 9 | 13 | 12 | 19 | 7 | 10 | 10 | 11 | 14 |
| | 0·5 | 48 | 40 | 12 | 7 | 12 | 10 | 11 | 5 | 4 | 7 | 11 | 15 |
| | 0·5 | 60 | 46 | 7 | 3 | 5 | 7 | 9 | 3 | 2 | | 6 | 13 |

47 surviving mice out of 50 injected with $CCl_4$ (1·0 ml/kg) had no lesions in liver or kidneys after 12 months.

90 surviving mice out of 100 without any treatment had no lesions in liver or kidneys after 12 months.

* 50 mice in each group at beginning of experiment.

teristics being different from normal tubular cells, in that the cytoplasm was more deeply staining, and the nuclei were larger and frequently in mitosis (Fig. 4). Other lesions, either unilocular or multilocular, had similar epithelia, were larger, up to 1·5 mm diameter, and had the epithelium thrown up into papillary folds.

*Distribution of lesions in the liver and kidneys*

The number of lesions seen in the liver and kidneys is set out in the Table. Tumours were counted macroscopically and, since they were readily visible, the figures represent the real number of lesions. However, "focal proliferations" in the liver and "papillary cysts" in the kidneys were in general not visible. The incidence in the liver was assessed by counting the number of lesions present in about 1 $cm^2$ area of random sections from each mouse. The incidence of kidney "papillary cysts" was determined by counting the number of lesions seen on screening the full sections of each kidney. Since the chance of any one 5$\mu$m section passing through a lesion of diameter 0·11–2·0 mm in a few random sections of an organ must be small, the actual number of "focal proliferations" must be larger than the counts made. The counts can only represent a measure of the chance of finding such a lesion, but are probably reasonable as measurable parameters of the incidence of the lesions.

*Liver tumours and "focal proliferations"*

The number of liver tumours in the surviving mice treated with DEN (80 mg/kg) varies significantly between the groups ($\chi$=31·3, 3 d.f., $P<0·001$). This is clearly due to the increased number of tumours in the groups given DEN 24 or 48 h after a dose of $CCl_4$, since the yield 60 h after $CCl_4$ is obviously not significantly different from that in the control group given no $CCl_4$. A similar variation is seen in the number of "focal proliferations". The increased number of liver tumours in the 24h-after-$CCl_4$ group is not significantly greater than in the 48h group ($\chi^2$=3·22, 1 d.f., N.S.); however, the yield of focal proliferations is also greater, and if the 2 parameters are added the increase is significant ($\chi^2$=13·2, 1 d.f., $P<0·001$).

The mice treated with 11 mg DMN/kg also show a significant variation in the yield of tumours between the groups ($\chi^2$=7·2, 3 d.f., $P<0·05$) due to the increased yield in the groups given $CCl_4$ 24 or 48 h previously. There is also a significant increase in the number of focal proliferations in the same 2 groups ($\chi^2$=8·5, 3 d.f., $P<0·05$); however the greater yield at 24 h than at 48 h is not statistically significant. Animals treated with 5 mg DMN/kg show a significant yield of tumours, which is greater in the animals dosed 24 h after $CCl_4$ but the yields are too small for statistical evaluation; the number of focal proliferations follows a similar course.

The results with animals given MEN show a similar trend. The overall yield of tumours is significant ($\chi^2$=6·6, 1 d.f., $P<0·01$) and is greater in mice treated 24 or 48 h after a dose of $CCl_4$, but not significantly so ($\chi^2$=4·45, 3 d.f., N.S.). A similar, but not statistically significant, trend is seen in the yield of "focal proliferations" ($\chi^2$=6·3, 3 d.f., $P\sim0·1$). If the 2 parameters are added, the increase at 24 and 48 h after a dose of $CCl_4$ is significant ($\chi^2$=12·1, 3 d.f., $P<0·01$).

As is the case for similar lesions in the rat (Pound *et al.*, 1973) the number of focal proliferations in a group appears to correlate with the number of tumours.

*Kidney lesions*

Only DMN produces tumours in the kidneys when acting alone, and then only at the higher dose. This is associated with a significant yield of papillary cysts ($\chi^2$= 115, $P<0·001$) at either dose level. A dose of $CCl_4$ 24 h beforehand increases the number of tumours produced by MEN ($\chi^2$=9·1, 1 d.f., $P<0·01$) and DMN ($\chi^2$= 4·11, 1 d.f., $P<0·05$) but not by DEN. The yields of tumours at the 48h and 60h intervals are not increased significantly. The number of papillary cysts is significantly increased by the previous dose of $CCl_4$ in the case of MEN ($\chi^2=15{\cdot}2$, 3 d.f., $P<0{\cdot}05$) but not with DEN ($\chi^2=5{\cdot}05$, 3 d.f., N.S.). There is obviously no significant variation from this cause in the case of DMN.

### DISCUSSION

Nitrosamines are potent carcinogens for kidney, liver and other tissues in rats, mice and other species (Magee and Barnes, 1967). This paper defines potencies for liver and kidneys in the local mouse strain, and supports the view that DEN is more potent for the liver and DMN for the kidney with the dose and conditions of these particular experiments. The susceptibilities of the liver and kidneys are not directly related, since, at effective dose levels, DEN produced more tumours in the liver than DMN and MEN, but few in the kidneys. MEN appears to follow a pattern of tumour induction similar to that of DMN rather than DEN, but tumour yields are too small for a rigorous assessment. This pattern is supported if the hepatic "focal proliferations" and the renal "papillary cysts" really are an early stage of neoplastic development (Pound *et al.*, 1973; Pound, unpublished).

The results confirm in mice the previous finding in rats (Pound *et al.*, 1973) that treatment with a single hepatonecrotic dose of $CCl_4$ 24 or 48 h before administration of DMN, increased the yield of tumours in the liver and kidneys, and that they show a similar effect when DEN and MEN are the carcinogens used. The number of hepatic "focal proliferations" was also increased in the case of DEN and DMN, and the same trend is evident with MEN, which adds to the impression that the number of these lesions correlated with the number of tumours. There was no increase with an interval of 60 h. This variation of the tumour yield with the interval between the doses of $CCl_4$ and nitrosamine shows that the number of tumours produced is greatest when the carcinogen is given during the period of most active regeneration after the dose of $CCl_4$ (*i.e.* from 24 to 72 h later; Hübner and Voigt, 1972). In the case of DEN, the increase is greatest when DNA synthesis is most rapid. These results are in accord with the increased susceptibility of the liver to many chemical carcinogens when regenerating actively after partial hepatectomy: *e.g.* to ethyl carbamate in mice (Pound, 1968*a*; Chernozemski and Warwick, 1970; Pound and Lawson, 1974*b*); 9 : 10 : dimethylbenz(a)anthracene in mice (Pound, 1968*a*; Marquardt, Sternberg and Phillips, 1970); DMN in rats (Craddock, 1971, 1975; Pound and Lawson, 1975*a*); DEN in rats (Grünthal *et al.*, 1970; Pound, unpublished); thioacetamide (Date, Gothoskar and Bhide, 1976) and other agents; although Craddock (1975) found no such increased susceptibility in the case of DEN.

The susceptibility of proliferating tissue to a carcinogen has also been demonstrated in the skin to some alkyl carbamates (Pound, 1966, 1968*a*; Pound and Withers, 1963; Hennings, Michael and Patterson, 1973), carcinogenic hydrocarbons (Pound, 1968*b*; Frei and Harsono, 1967) and UV light (Pound, 1970). It therefore appears to be a general phenomenon, and has raised the question whether the susceptibility may be restricted to a particular phase of the cell cycle, for example the S phase. However, unequivocal evidence of a precisely restricted susceptible period is not yet available in respect of the liver (Chernozemski and Warwick, 1970; Pound and Lawson, 1974*b*) nor the skin (Hennings *et al.*, 1973; Pound, unpublished). The experiments in this paper were not designed to elucidate this point.

After surgical removal or chemical destruction of part of the liver, the activities of many microsomal enzymes in the remaining liver, associated with the metabolism of many chemicals, are reduced within one hour, and the reduction persists for 3–4 days (Barker, Arcasoy and Smuckler, 1969; Henderson and Kersten, 1970, 1971). The level of activity of DMN demethylase is decreased (Pound and Lawson, 1974*b*, 1975*a*, *b*) and the metabolic elimination of DMN by the liver is slowed (Craddock, 1971) so that the tissues are exposed to the chemical for a longer time. An enzyme of this nature may be an index of the activity of, or may actually be involved in the formation of the active carcinogenic metabolite of, the nitrosamines and, since all such enzymes may not be influenced in the same way, the increased tumour yield might be contributed to by alterations in the metabolic pathways. Such factors may account for the increase in the number of kidney tumours in mice found after the dose of $CCl_4$, and in rats given DMN or DEN after partial hepatectomy (Rabes, Hartenstein and Gminder, 1971; Meister and Rabes, 1973; Pound *et al.*, 1973; Pound and Lawson, 1975*b*) but only partly, since the period of increased susceptibility starts later and is less persistent than the period during which the microsomal enzymes are inactivated.

Alterations in the metabolic pathways might also influence the type and site of binding to DNA. Alkylation of nucleic acids has been reviewed recently (Singer, 1975). Alkylation of the $O^6$ position of guanine (O'Connor, Capps and Craig, 1973) and esterification of phosphate groups by DMN (O'Connor, Margison and Craig, 1975) have been demonstrated. In the case of ethyl carbamate, the only type of binding to DNA that has been demonstrated has been the formation of DNA phosphate esters (Pound, Franke and Lawson, 1976) but no alteration was found after partial hepatectomy. The relative significance of the different types of binding in relation to the carcinogenic process is not known.

The nature of the "proliferative foci" (to use a descriptive term with no pathogenetic implications) is of importance. Similar lesions have been described in animals given various chemical carcinogens, and been referred to as preneoplastic nodules, hyperplastic nodules and other terms (Farber, 1973). A recent workshop has suggested that similar lesions might be called "foci of cell proliferation" (Squire and Levitt, 1975). Some of these terms have been used for the (hyperplastic) nodules in a cirrhotic liver, whether or not the causative agent is a carcinogen, and this is confusing. The proliferative foci of this paper and previously recorded (Pound *et al.*, 1973) occurred in livers that were histologically normal except for the occasional presence of an obvious tumour. It was suggested that these lesions might be the early stage of neoplastic growth because of their structure, manner of growth and the correlation between their number and the number of tumours, a view which will be reported in detail elsewhere (Pound, unpublished).

Lastly, attention is drawn to the "papillary cysts" in the kidneys of animals given the nitrosamines. The number of these also appears to be increased when the animals are dosed after a dose of $CCl_4$. Similar lesions have been reported as epithelial dysplasias in animals given DMN (McGiven and Ireton, 1972). It seems likely that these lesions have a relationship to the renal epithelial tumours similar to that of the focal proliferations in the liver to the hepatocellular tumours (Hard and Butler, 1971).

This work was supported by the Mayne Bequest Fund and the Tooth Bequest of the University of Queensland.

### REFERENCES

BARKER, E. A., ARCASOY, M. & SMUCKLER, E. A. (1969) A Comparison of the Effects of Partial Surgical and Partial Chemical ($CCl_4$) Hepatectomy on Microsomal Cytochrome $b_5$ and $P_{450}$ and Oxidative N-Demethylation. *Agents and Actions*, **1**, 27.

CHERNOZEMSKI, I. N. & WARWICK, G. P. (1970) Liver Regeneration and Induction of Hepatomas in $B6AF_1$ Mice by Urethan. *Cancer Res.*, **30**, 2685.

CRADDOCK, V. M. (1971) Liver Carcinomas Induced in Rats by Single Administration of Dimethylnitrosamine after Partial Hepatectomy. *J. natn. Cancer Inst.*, **47**, 899.

CRADDOCK, V. M. (1973) Induction of Liver Tumours in Rats by a Single Treatment with Nitroso Compounds given after Partial Hepatectomy. *Nature, Lond.*, **245**, 386.

CRADDOCK, V. M. (1975) Effect of a Single Treatment with the Alkylating Carcinogens Dimethylnitrosamine, Diethylnitrosamine and Methyl methane-sulphonate, on Liver Regenerating after Partial Hepatectomy. I. Test for Induction of Liver Carcinomas. *Chem.-Biol. Interactions*. **10**, 313.

DATE, P. A., GOTHOSKAR, S. V. & BHIDE, S. V. (1976) Effect of Partial Hepatectomy on Tumour

Incidence and Metabolism of Mice Fed Thioacetamide. *J. natn. Cancer Inst.*, **56**, 493.

DUTTON, A. H. & HEATH, D. F. (1956) The Preparation of [$^{14}$C]Dimethylamine and [$^{14}$C]Dimethylnitrosamine. *J. Chem. Soc.*, 1892.

FARBER, E. (1973) Hyperplastic Liver Nodules. In *Methods in Cancer Research, Vol. VII*, Ed. H. Busch. New York: Academic Press. p. 345.

FREI, J. V. & HARSONO, T. (1967) Increased Susceptibility to Low Doses of a Carcinogen of Epidermal Cells in Stimulated DNA Synthesis. *Cancer Res.*, **27**, 1482.

GRÜNTHAL, D., HELLENBROICH, D. O., SÄNGER, P. & MAASS, H. (1970) Der Einfluss von partiellen Hepatektomien auf die Hepatomrate nach Diäthylnitrosamin-Gaben. *Z. Naturforsch.*, **25**, 1277.

HARD, G. C. & BUTLER, W. H. (1970) Cellular Analysis of Renal Neoplasia: Induction of Renal Tumours in Dietary-conditioned Rats by Dimethylnitrosamine, with a Reappraisal of Morphological Characteristics. *Cancer Res.*, **30**, 2796.

HARD, G. C. & BUTLER, W. H. (1971) Morphogenesis of Epithelial Neoplasms Induced in the Rat Kidney by Dimethylnitrosamine. *Cancer Res.*, **31**, 1496.

HENDERSON, P. T. & KERSTEN, K. J. (1970) Metabolism of Drugs During Rat Liver Regeneration. *Biochem. Pharmac.*, **19**, 2343.

HENDERSON, P. T. & KERSTEN, K. J. (1971) Alteration of Drug Metabolism during Rat Liver Regeneration. *Archs int. Pharmacodyn. Thér.*, **189**, 373.

HENNINGS, H., MICHAEL, D. & PATERSON, E. (1973) Enhancement of Skin Tumorigenesis by a Single Application of Croton Oil before or Soon after Initiation by Urethan. *Cancer Res.*, **33**, 3130.

HÜBNER, K. & VOIGT, H. G. (1972) Histoautoradiographische Untersuchungen zur Leber-regeneration nach $CCl_4$-Vergiftung. *Med. Welt.*, **23**, 1348.

LAWSON, T. A. & POUND, A. W. (1973) The Interaction of Carbon-14-Labelled Alkyl Carbamates, Labelled in the Alkyl and Carbonyl Positions, with DNA *In vivo*. *Chem.-Biol. Interactions*, **6**, 99.

MAGEE, P. N. & BARNES, J. M. (1967) Carcinogenic Nitroso Compounds. *Adv. Cancer Res.*, **10**, 163.

MCGIVEN, A. R. & IRETON, H. J. C. (1972) Renal Epithelial Dysplasia and Neoplasia in Rats given Dimethylnitrosamine. *J. Path.*, **108**, 187.

MARQUARDT, H., STERNBERG, S. S. & PHILLIPS, F. S. (1970) 7,12-Dimethylbenz(a)anthracene and Hepatic Neoplasia in Regenerating Rat Liver. *Chem.-Biol. Interactions*, **2**, 401.

MEISTER, P. & RABES, H. (1973) Nierentumoren durch Diathylnitrosamin nach partieller Leberresektion: Morphologie und Wachstumsverhalten. *Z. Krebsforsch.*, **80**, 169.

O'CONNOR, P. J., CAPPS, M. J. & CRAIG, A. W. (1973) Comparative Studies of the Hepatocarcinogen N,N-dimethylnitrosamine *In vivo*: Reaction Sites in Rat Liver DNA and the Significance of their Relative Stabilities. *Br. J. Cancer*, **27**, 153.

O'CONNOR, P. J., MARGISON, G. P. & CRAIG, A. W. (1975) Phosphotriesters in Rat Liver Deoxyribonucleic Acid after the Administration of the Carcinogen NN-dimethylnitrosamine *In vivo*. *Biochem. J.*, **145**, 475.

POUND, A. W. (1966) Further Observations Concerning the Influence of Preliminary Stimulation by Croton Oil and Acetic Acid on the Initiation of Skin Tumours in Mice by Urethane. *Br. J. Cancer*, **20**, 385.

POUND, A. W. (1968*a*) Carcinogenesis and Cell Proliferation. *N.Z. med. J.* (*Special Issue*), **67**, 88.

POUND, A. W. (1968*b*) The Influence of Preliminary Initiation by Acetic Acid or Croton Oil on Skin Tumour Production in Mice after a Single Application of Dimethylbenzanthracene, Benzopyrene or Dibenzanthracene. *Br. J. Cancer*, **22**, 533.

POUND, A. W. (1970) Induced Cell Proliferation and the Initiation of Skin Tumour Formation in Mice by Ultraviolet Light. *Pathology*, **2**, 269.

POUND, A. W., FRANKE, F. & LAWSON, T. A. (1976) The Binding of Ethyl Carbamate to DNA of Mouse Liver *In vivo*: The Nature of the Bound Molecule and the Site of Binding. *Chem.-Biol. Interactions*, **14**, 149.

POUND, A. W. & LAWSON, T. A. (1974*a*) Effects of Partial Hepatectomy on Carcinogenicity, Metabolism, and Binding to DNA of Ethyl Carbamate. *J. natn. Cancer Inst.*, **53**, 423.

POUND, A. W. & LAWSON, T. A. (1974*b*) Protection by a small Dose of Carbon Tetrachloride against the Toxic Effects of Dimethylnitrosamine in Rats. *Br. J. exp. Path.*, **55**, 203.

POUND, A. W. & LAWSON, T. A. (1975*a*) Protection by Carbon Tetrachloride Against the Toxic Effects of Dimethylnitrosamine in Mice. *Br. J. exp. Path.*, **56**, 77.

POUND, A. W. & LAWSON, T. A. (1975*b*) Partial Hepatectomy and Toxicity of Dimethylnitrosamine and Carbon Tetrachloride in Relation to the Carcinogenic Action of Dimethylnitrosamine. *Br. J. Cancer*, **32**, 596.

POUND, A. W., LAWSON, T. A. & HORN, L. (1973) Increased Carcinogenic Action of Dimethylnitrosamine after Prior Administration of Carbon Tetrachloride. *Br. J. Cancer*, **27**, 451.

POUND, A. W. & WITHERS, H. R. (1963) The Influence of Some Irritant Chemicals and Scarification on Tumour Initiation by Urethane in Mice. *Br. J. Cancer*, **17**, 460.

RABES, H., HARTENSTEIN, R. & GMINDER, J. (1971) Nierentumoren durch Diaethylnitrosamin bei partiell hepatektomierten Ratten. *Naturwissenschaften*, **58**, 102.

RIOPELLE, J. L. & JASMIN, G. (1969) Nature, Classification, and Nomenclature of Kidney Tumors Induced in the Rat by Dimethylnitrosamine. *J. natn. Cancer Inst.*, **42**, 643.

SINGER, B. (1975) The Chemical Effects of Nucleic Acid Alkylation and their Relation to Mutagenesis and Carcinogenesis. In *Progress in Nucleic Acid Research and Molecular Biology Vol.* **15**. Ed. W. E. Cohn. New York: Academic Press Inc., p. 219.

SQUIRE, R. A. & LEVITT, M. H. (1975) Report of a Workshop on Classification of Specific Hepatocellular Lesions in Rats. *Cancer Res.*, **35**, 3214.